# The Progress of Medical Image Semantic Segmentation Methods for Application in COVID-19 Detection

**DOI:** 10.1155/2021/7265644

**Published:** 2021-11-22

**Authors:** Amin Valizadeh, Morteza Shariatee

**Affiliations:** ^1^Department of Mechanical Engineering, Ferdowsi University of Mashhad, Mashhad, Iran; ^2^Department of Mechanical Engineering, Iowa State University, Ames, IA, USA

## Abstract

Image medical semantic segmentation has been employed in various areas, including medical imaging, computer vision, and intelligent transportation. In this study, the method of semantic segmenting images is split into two sections: the method of the deep neural network and previous traditional method. The traditional method and the published dataset for segmentation are reviewed in the first step. The presented aspects, including all-convolution network, sampling methods, FCN connector with CRF methods, extended convolutional neural network methods, improvements in network structure, pyramid methods, multistage and multifeature methods, supervised methods, semiregulatory methods, and nonregulatory methods, are then thoroughly explored in current methods based on the deep neural network. Finally, a general conclusion on the use of developed advances based on deep neural network concepts in semantic segmentation is presented.

## 1. Introduction

Semantic segmentation of medical images is also known as pixel-level classification. The task is to cluster the parts of the image side by side, which belong to a class of similar objects [1]. The other two key functions of the image are to classify the image's surface and define it. Image classification ensures that each image is exchanged as an equal group of images of similar groups, and monitoring also refers to the object's location and recognition. For predicting pixel level, image segmentation can be used as it categorizes each pixel. Furthermore, there is a task that identifies and separates joints called sample segmentation [2, 3]. Medical image semantic segmentation has a variety of applications, such as road sign detection [4], colon crypt segmentation [5], land-use classification, and land surface classification [6]. It is also widely used in medicine, such as brain and tumor detection [7] and discovering and tracking medical devices in surgery [8]. Numerous applications of segmentation in medicine are listed in some studies [8]. Scene resolution is of great significance in advanced driver assistance systems (ADAS) or car driving areas and depends extensively on semantic image segmentation ([9–11]). Research has developed a deep-learning (DL-) based system for assessing disease. This system automatically scans the location of the disease and measures the shape, size, and percentage of the disease on the CT image of people who have COVID-19 disease. In this study, a strategy (HITL) was proposed for the repeated production of training samples. This method is for radiologists to evaluate the results of DL segmentation, make changes, and frequently add more tutorials to update the model. As a result, they speed up the algorithm's development cycle [12]. As recent findings show, before choosing to use chest CT, a significant number of imaging studies need to be checked for patient diagnosis or patient screening. Artificial intelligence technology, particularly DL analysis tools, could potentially be created to support radiologists in triage, quantification, and data analysis. Artificial intelligence solutions can analyze several cases to determine if a chest CT scan shows lung abnormalities. If the software significantly increases the risk of developing the disease, the case will be reviewed by a radiologist or a physician for further treatment/quarantine. Such systems or their modifications, after validation and testing, can be a key factor in the diagnosis and control of patients with the virus [13]. The pandemic of COVID-19 appears to have negative impacts on world health and well-being. An important method in combating COVID-19 is efficient screening in infected patients, and one of the most important screening methods is radiological test utilizing chest radiography. Preliminary studies have shown that patients infected with COVID-19 have problems with chest radiographs. COVID-Net is implementing a deep concealer neural network architecture to diagnose COVID-19 patients implying chest X-ray (CXR) images motivated by the academic community's open-source endeavors. This information is accessible to the public [14]. In the context of e-healthcare, Zhang et al. showed a privacy-preserving optimization of the clinical pathway query method (PPO-CPQ) [15]. Ala et al. have used a metaheuristic algorithm and optimized an appointment scheduling issue for healthcare systems depending on the quality of fairness service [16]. Also, Xu et al., to simulate pathogenesis diagnosis, proposed a computer technique called network differentiation [17]. Segmentation accuracy has greatly improved since the reemergence of the deep neural network. In general, traditional methods are called the methods that came before the deep neural network. The following parts of this convention are followed in this study and standard segmentation techniques are briefly analyzed in this article, and, most significantly, this development builds on the recent progress of adopting and organizing a deep neural network from different aspects. Furthermore, the image segmentation measurement and assessment databases are checked. The rest of this study is organized as follows: In the dataset and assessment criteria, Section 2 explores the semantic segmentation of the image. A description of traditional methods is given in Section 3.  Section 4 outlines recent developments in detail. Finally, a description of the work performed is given with conclusions in Section 5.

## 2. Datasets and Evaluation Metrics

### 2.1. Datasets

**Figure 1:**
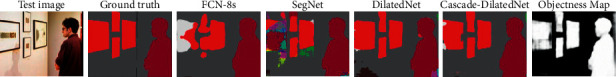
Examples of ADE20K data images. From left to right and from top to bottom, the first segmentation of object masks is seen. The second to fifth elements of photo segmentation are linked to the object's portions (e.g., body parts, glass parts, and photo board parts). In the sixth segment, parts of the head are displayed (such as the eyes, mouth, and nose) [17].

Many general datasets are currently connected to image segmentation, such as PASCAL VOC, MS COC, ADE20K, and, in the field of the autonomous driving area, Cityscapes [11] and KITTI ([9, 10]). The challenge of visual object classes, or VOCs, consists of two components [15]: (1) image and annotation datasets that are available to the public and (2) annual workshops and competitions that are held online on some websites and sometimes in person. The main challenges have been dealt with since 2005. By 2012, the challenge included 20 classes. Educational and validation data contained 11530 images containing 27450 annotated objects with areas of interest and 6929 segmented images. Also, in image segmentation, datasets have been extensively utilized. The Microsoft COCO dataset [2] contains images of 91 objects, where a 4-year-old person can quickly identify with 2.5 million labeled samples in 328,000 images. Authors also introduced the dataset with a detailed statistical analysis compared to PASCAL data [15], ImageNet data [18], and SUN data [19]. An analysis of 50 chest X-ray images of 25 positive COVID-19 cases was confirmed because of the lack of an available COVID-19 dataset. Seven distinct architectures from neural network models are used in COVIDX-Net. Each deep neural network model can analyze the number of X-ray images to identify the patient's state as negative or positive COVID-19 [20]. The authors in [20] collected images from 5 different sources to test this idea and generate a dataset of 170 X-ray images and 361 CT images of COVID-19. Two explanations exist for the use of photographs from these sources. First, to help radiologists diagnose COVID-19 worldwide, it is important to design an advanced tool. Second, for the scientific community and the general public, photographs of these sources are openly available. Also, the images used in it will be publicly available in a GitHub repository [21]. With 150 classes of objects and materials, ADE20K data [17] is another scene analysis criterion. ADE20K data contains the object segmentation mask and component segmentation mask, unlike other datasets. There are also several pictures of parts of the head (like the mouth, eyes, and nose). In the training suite, there are precisely 20210 images, and there are 2,000 images in the validation suite and 3,000 images in the experimental suite [17]. Some of these images are depicted in Figure 1.

**Figure 2:**
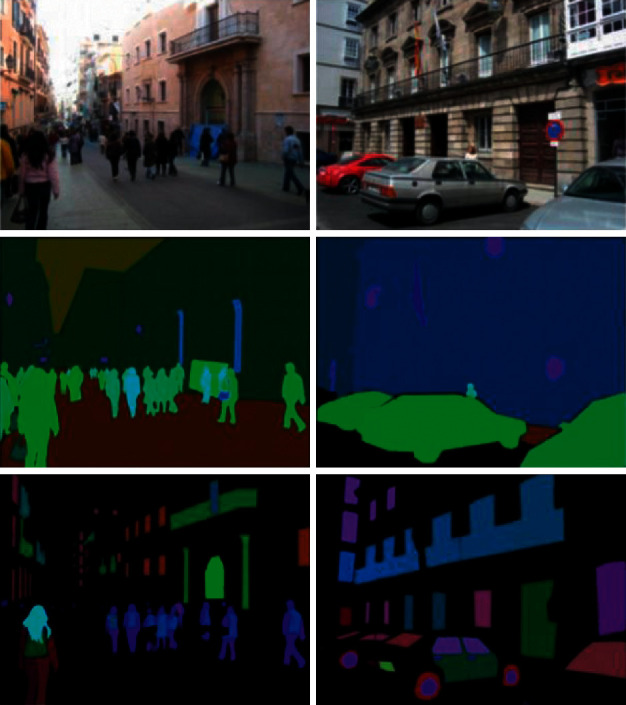
One of the images in the Cityscapes database [17].

The Cityscapes dataset is a criterion that focuses on understanding the meaning of urban street scenes [11]. The collection contains 30 groups obtained from 50 towns in 5,000 fine annotated pictures. The selection period, which includes spring, summer, and autumn, is also several months.  Figure 2 displays one of the images of this data in the annotations.

**Figure 3:**
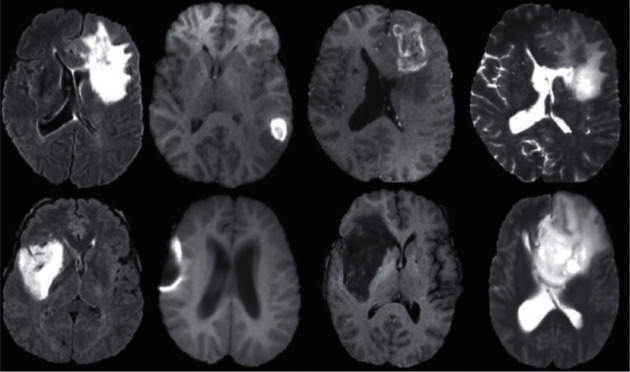
Example of BraTS dataset [24].

The KITTI dataset [9], another autonomous driving dataset recorded by driving on highways and in rural areas around Karlsruhe, is another example of semantic image data. On average, a maximum of 15 cars and 30 pedestrians can be seen in each image. Zhou et al. proposed a model for evaluating the clarity of screen content and natural scene images while blind [10]. Lv et al. proposed a deep-learning-based fine-grained visual computation [11]. Liu et al. have investigated the Style and Characters Inpainting Based on CGAN [12]. Road detection, stereo reconstruction, light current visual measurement, 3D object detection, and 3D tracking are the principal functions of this dataset (http://www.cvlibs.net/datasets/kitti/). One use of image segmentation is in automated vehicles. The system uses augmented reality to describe the amount of automation and its dependability to increase the system's confidence and reliability. In addition to the above databases, there are many others such as SUN, the Visual Database of Shadow Detection or Texture Segmentation (https://zenodo.org/record/59019#.WWHm3oSGNeM), Berkeley segmentation dataset [22], and LabelMe dataset [23] whose complete information can be found (http://homepages.inf.ed.ac.uk/rbf/CVonline/Imagedbase.htm). There are various imaging models in the field of medical data, most of which have been applications of DL methods on MRI, mammography, or CT scan imaging data. However, different areas of the body have this data or even other imaging samples. However, the main focus of this research is on three datasets, which are from the brain and chest areas. One of the most important brain MRI datasets working on diagnosing tumors, Alzheimer's, and MS is BraTS dataset (https://ieee-dataport.org/competitions/brats-miccai-brain-tumor-dataset). From the download path of this dataset, there will be four folders: T1, T2, Flair, and T1Ce, respectively. Each section has 155 sections, MRI image sections are weak, and there are 155 sections for a dataset and 210 sections for a high-grade Glioma dataset. At the same time, 75 sections are in another type of Glioma. Hence, the number is 285 cases. This dataset has versions between 2012 and 2019, and an example of the images of this dataset is in form (3). There is also another original dataset called TCGA-GBM, which has higher quality 3D images than BraTS (https://portal.gdc.cancer.gov/projects/TCGA-GBM) (see Figure 3).

There are also valid datasets in the field of mammography images used to diagnose breast tumors, one of the most important of which is a dataset called MIAS (https://www.mammoimage.org/databases).

**Figure 4:**
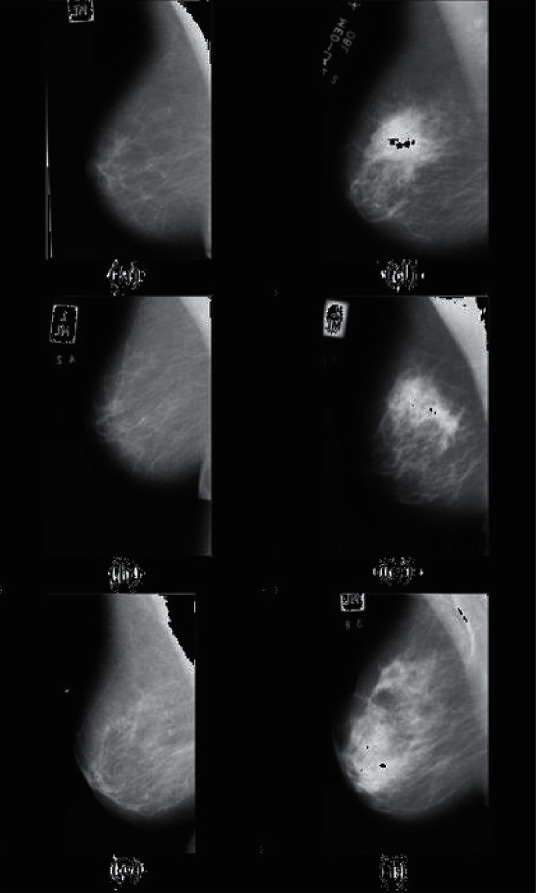
Sample image of normal and malignant tumor based on MIAS dataset [25].

Also, on the same website, other datasets called DDSM, AMDI, and IRMA are used to diagnose cancerous tumors in different shapes and sizes as benign and malignant. An example of these images is shown in Figure 4.

### 2.2. Evaluation Metrics

For image segmentation and scene analysis, standard performance assessment metrics include pixel resolution *P*_acc_ , middle resolution *M*_acc_, region intersection upon the union *M*_IU_, and connection area sharing frequency weight FW_IU_. It is presumed that *n*_*ij*_  describes the number of class *i* pixels that are supposed to belong to class *j*, where there are various groups *n*_*d*_ and it is assumed that *t*_*i*_=∑_*J*_*n*_*ij*_  represents the number of pixels in class *i*. All of these relationships are written in the four following formulas [26]:

(1) $$\begin{array}{c} P_{\text{acc}}=\frac{\sum_{i}n_{ii}}{\sum_{i}t_{i}}, \end{array}$$

(2) $$\begin{array}{c} M_{\text{acc}}=\frac{1}{n_{cl}}\sum_{i}\frac{n_{ij}}{t_{i}}, \end{array}$$

(3) $$\begin{array}{c} M_{\text{IU}}=\frac{1}{n_{cl}}\sum_{i}\frac{n_{ij}}{t_{i}+\sum_{j}n_{ji}-n_{ii}}, \end{array}$$

(4) $$\begin{array}{c} {\text{FW}}_{\text{IU}}=\frac{1}{\sum_{k}t_{k}}\sum_{i}\frac{t_{i}n_{ii}}{t_{i}+\sum_{j}n_{ji}-n_{ii}}. \end{array}$$

There are also other evaluation metrics for the segmentation of medical images, which are popularly used in scientific societies. These include accuracy, sensitivity, specificity, recall, ROC curve, and Area under Curve (AUC) rate.

## 3. Traditional Methods

Before presenting deep neural networks, features and classification methods are applied to the most important topics [19]. A feature is a piece of data that is applied to solving computational tasks in machine vision and image processing. That is the same context of machine learning precision and the identification of patterns [27]. For semantic segmentation in images, a number of features are used, such as pixel color, histogram of oriented gradients (HOG) ([28]), scale-invariant feature transform (SIFT) [29], local binary pattern (LBP) [30], SURF method [31], Harris Corner Detection [32], method of Shi-Tomasi [33], subpixel corner method [34], SUSAN edge method [35], Features from Accelerated Segment Test (FAST) [36], FAST-ER method [37], AGAST method [38], AGAST multiscale detection method [39], the bag-of-visual-words (BOV) [40], the Poselets method [41], the Textons method [42], and many other methods. Approaches to image semantic segmentation do not include supervised or unsupervised cases [43]. In particular, thresholding, which is commonly used in gray surface images, is a simple method. In the medical industry, optimization, classification, and diagnosis are very common, using imaging equipment ([44–46]). In general, in this regard, thresholding methods are very efficient. K-means clustering means an unsupervised clustering process. The K-means algorithm specifies that the number of clusters must be defined in advance. The points of *k* are initially randomly positioned in the property space. Additionally, each datum is allocated to the closest points. The gravity points are then successively transferred to the middle of the cluster. This process proceeds until the stop criterion is met [47]. The problem of segmentation can be used as an energy model, resulting from a compression method [48]. Intuitively, edges are an important part of segmentation, and there is also much research on edge recognition ([49–53]). Also, edge-based approaches and regional growth methods are other branches. Support vector machines (SVMs) are binary classifiers that are well studied and are employed in many jobs. Inseparable linear problems can be solved with the slack variables, too [54, 55]. The core approach was also used for integral tasks by the mapping of dimensional broader features. A Markov random field (MRF) is a set of randomized variables with an indirect diagram of a Markov attribute. Also, the Markov stochastic grid is a directionless graphical model (http://host.robots.ox.ac.uk/pascal/VOC/voc2010/results/index.html; [56]).

In general, a case study has been made between the methods of segmentation in images in the field of semantic segmentation, which is shown in Table 1. Classic studies on the detection of cancerous tumors from MRI images have also been presented. In [57], the method of Brownian motion of water molecules to produce contrast has been done. Also, in [58] an improved edge detection method for segmentation is presented. The watershed method and the hierarchical clustering algorithm have also been studied in [59]. Also, in [60], anisotropic diffusion based on segmentation and pattern based on group classification based on support vector machine and segmentation with FCM has been done. The application of a genetic algorithm and discrete wavelet transform thresholding method is presented in [59]. Qiao et al. have presented a local wavelet acoustic pattern and an MLP optimized by a modified Whale Optimization algorithm for classification of underwater objects [59].

Also, [65] presents a combined approach called ant colony optimization (ACO) algorithm and genetic algorithm. In [66], the chaotic firefly algorithm based on the FCM algorithm has been performed. The application of the optimal Particle Swarm Optimization (PSO) in [67] has also been studied, and in [68] a bat optimization algorithm for segmenting MRI images for different purposes is presented. In general, a case comparison between the existing methods in the segmentation field in MRI images has been done, which is shown in Table 2.

In the field of classical methods used to segment mammographic images, we can refer to the research [69] that has used the segmentation method of regional growth with a cellular neural network with a specific threshold. The use of a Back-Propagation (BP) neural network has also been considered in [70]. Applying the new Naïve Bayesian method in [71] has also been considered in this field. In [72], the regression-based evolutionary methods are used to diagnose breast cancer tumors to estimate and predict the remaining life based on the size of the tumor. In [73], the classification or diagnosis of breast cancer in mammographic images combined with wavelet analysis and genetic algorithm is presented. Xu et al. provided a method for identifying, classifying, and predicting nucleic acid-binding proteins [74].

Also, [75] presents a semisupervised adaptive algorithm named GrowCut for the segmentation of tumors of interest areas or ROI of mammographic images based on the amendment of the law of automatic evolution. In general, a case comparison has been made between the methods available for segmentation in mammographic images, which is shown in Table 3.

## 4. Recent Deep Neural Network Methods in Segmentation

### 4.1. Artificial Neural Network (ANN)

**Figure 5:**
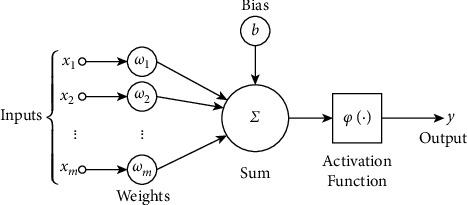
Model of artificial neurons [80].

Biological neurons are inspired by the artificial neural network (ANN). An artificial neuron is an essential element of an artificial neural network. Each artificial neuron has only inputs that weigh. Neurons issue a scale following a transfer function or activation function. An instance of a neural model is shown in Figure 5.

**Figure 6:**
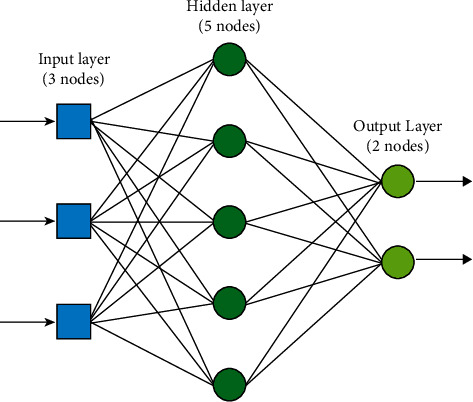
An example of a model of an artificial neural network [85].

Based on artificial neurons, the accumulation of different neurons is automatically Autoencoder [81], Restricted Boltzmann Machine (RBM) [82], Recurrent Neural Network (RNN) or convolutional neural network (CNN) [83], Long Short-Term Memory (LSTM) [84], and other models. The basic architecture is shown in Figure 6.

A shared weight architecture, influenced by biological mechanisms, is used by the convolutional neural network (CNN) [83]. The connection pattern between neurons mimics the development of the visual cortex of the animal. Acceptance is another essential term, indicating that individual cortical neurons can respond to stimuli only in a small region of the visual field. They also have immutable or complex spatial properties dependent on the architecture with shared weight and spatial characteristics. Due to this excellent structure, the convolutional neural network has gained significance which caused image classification, segmentation, and detection. In the following section, recent developments using convolutional neural networks in the semantic segmentation of the image will be presented.

### 4.2. Fully Convolutional Network (FCN)

**Figure 7:**
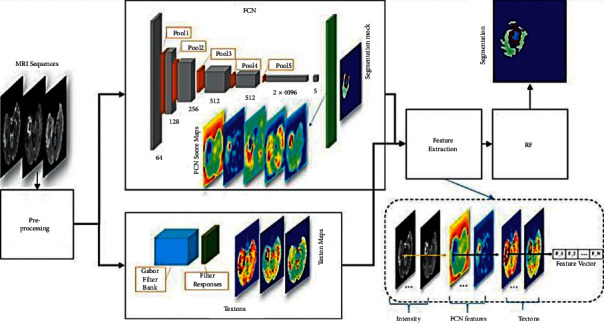
FCN architecture [87].

The article in [86] represents the first study in the image segmentation field to present ANNFCN. Replacing the utterly connected layer with the fully convolutional layer is the fundamental concept of this method. Using the interpolation layer, the network recognizes that the output size is just like the input required for segmentation. Most significantly, by successful inference and learning, the network is educated, takes on the required size, and produces the correct size output. The FCN was introduced in VGG-Net and has reached a substantial role in the segmentation of PASCAL VOC (20 percent relative increase to 62.2 percent of the average IU in 2012). However, the assumption takes less than one-fifth of a second for a regular image. The main FCN architecture is shown in Figure 7.

### 4.3. Interpolation against Parsing in Medical Image Semantic Segmentation

**Figure 8:**
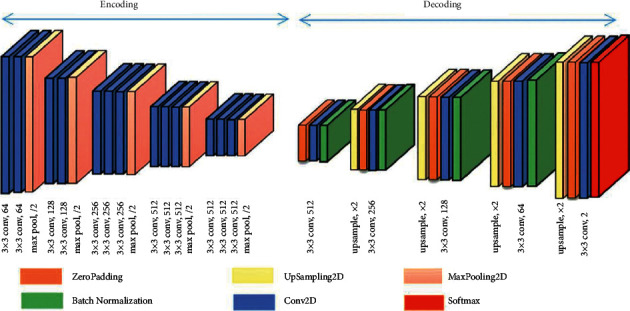
Convolution decomposition network architecture [91].

The parsing layer is also approved in the semantic segmentation of images and the FCN architecture. Degradation and sampling layers in the pooling layer that define pixel type labels and predict segmentation masks are the degradation network used in [88]. Unlike FCN in [88], this grid is used for proposed thing designs to get the synthetic parts as an example for the final semantic segmentation. The sampling method step adopts two-line interpolation, which can be found in [86]. The sampling stage of the samples has commonly approved two-line interpolation due to the computational efficiency and good retrieval of the original image. The decomposition operation is an inverse calculation of the convolution function that can also retrieve the input size. It can then be utilized to segment the function mapping size to the original input size to retrieve it. The architecture used in [88] is seen in Figure 8 . Some researchers still use the decomposition layer in multiple versions to introduce semantic segmentation, which can be found in [74, 89, 90].

CT images are also used to obtain information about COVID-19 patients. The CT image shows the condition of the patients' lungs and shows how much the disease has affected the lungs.

### 4.4. Connect FCN with CRF and Other Traditional Methods

Responses in the last layer of deep convolutional neural networks (DCNNs) are not sufficiently localized to effectively segment an entity, according to DeepLab research [92]. Mixing an ultimately linked random field or CRF in the DCNN end layer solves this poor localization property. Authors' method in the test determined in the semantic image segmentation work of PASCAL VOC-2012 reaches 71.6% IOU accuracy. After this, they implement another segmentation architecture by matching Domain Transform (DT) with DCNN [92]. Since the dense CRF inference is costly in terms of computation, Domain Transform (DT) relates to a modern method of maintaining edge filtering. A reference edge mapping governs the smoothing rate. The Domain Transform (DT) is several times more rapid than the dense CRF assumed. Finally, studies compare the effects of semantic segmentation and reliably document the boundaries of the object. Researchers also use superpixels to segment images in the domain of the semantic segmentation [93]. Reference [94] deals with semantic segmentation by merging rich details, including mixing label fields and high-order relationships, in the Markov random field (MRF).

### 4.5. Dilated Convolution

**Figure 9:**
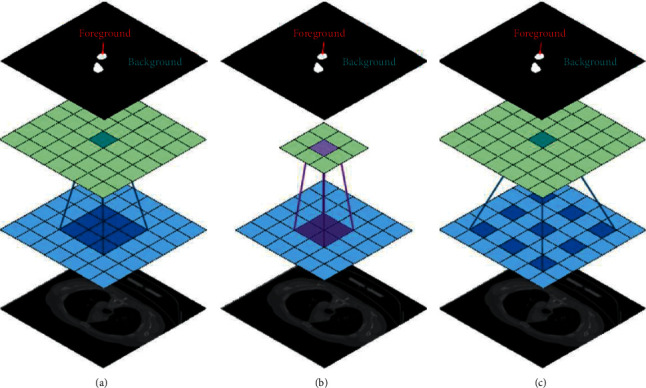
An example of an available convolution structure (Atrous convolution or hole convolution). (a) Convolution layer with 3 × 3 core size; a normal displacement operation with expansion parameter-1, (b) open convolution with expansion parameter-2, and (c) open convolution with expansion parameter-3 [95].

The majority of semantic segmentation methods focus on the compatibility of convolutional neural networks (CNNs), initially designed to classify images. Dense prediction, however, is structurally distinct from classification, as are semantic image segmentation tasks. An instance of an open structure of convolution can be seen in Figure 9. Reference [92] has previously used this technique in its work, which is named Atheros convolution or convolution hole [92] or open convolution [96]. Convolution was originally developed to efficiently calculate wavelet transform in an “algorithm à trous” scheme [97]. Reference [96] systematically presented a module using discrete complexities for collecting multiscale textual information. This architecture is based on open convolution, which supports the expansion of the exponential receiver field without losing sharpness or coverage, since the available convolution contains networked (or segmented) network artifacts in the input data.

Reference [98] created an approach called dilated residual networks (DRN) to eliminate these artifacts and further improve network efficiency.

### 4.6. Progress in the Main Pillar of the Network

The network's main column refers to the network's main structure. As it turns out from image classification tasks, the key source of image medical semantic segmentation is derived. The FCN [86] approved the VGG-16 net structure [99], which performed exceptionally well in ILSVRC14. Authors also considered the architecture of AlexNet [100], which won ILSVRC12, as well as GoogLeNet [101], and performed well in ILSVRC14. The VGG network has been validated in many previous studies [92, 94]. Following the release of ResNet or the deep residual network [102], DeepLab took its place in the ILSVRC 2015 classification work and implemented it, and semantic segmentation has made new progress. To reach a sufficient configuration, [103] evaluates the various changes of a fully complex residual network, including feature mapping resolution, number of layers, and field of view size. Also, [104] examines the remaining deep networks and explains some of the experimentally observed habits. As a result, authors get a shallow network architecture that in the ImageNet classification dataset is dramatically better than much deeper ones. Recently, ResNeXt [105] was introduced as the next generation of ResNet. This network is the basis for entering the ILSVRC 2016 classification work, in which it has won second place. GoogleNet also acquires extensions such as Inception-v2, Inception-v3 [106], Inception-v4, and Inception-ResNet [101], which has already been approved in the article [107].

## 5. Pyramid Methods in Semantic Segmentation

Aside from adopting networks with strong core columns, researchers are also trying to combine a pyramid strategy with CNN. A clear example of that is a pyramidal structure.

### 5.1. Image Pyramid

**Figure 10:**
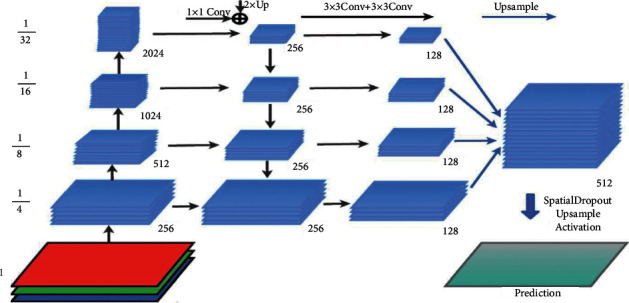
Three levels of the image pyramid [109].

An image pyramid [108] is a series of sequentially segmented images before any of the desired stop criteria has been met. There are two different types of image pyramids: the Gaussian pyramid used for image sampling and the Laplacian pyramid used to recreate a scattered image from the lower image (with lower resolution). Three levels of the image pyramid can be seen in Figure 10.

**Figure 11:**
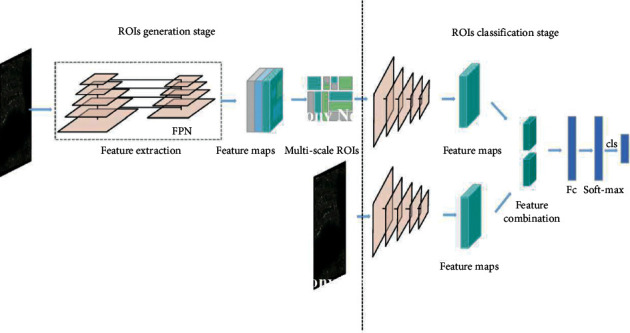
Image pyramid used in CNN structure [112].

In the field of image semantic segmentation, [110] establishes a network that can efficiently boost output with traditional multiscale image input and sliding pyramid mixing. This architecture captures the sense of the background patch. Similarly, by feeding input images of various sizes into a deep sharing network, DeepLab implements an image pyramid structure [111] that extracts multiscale functionality. The resulting features are combined for pixel classification at the end of each deep grid. You can see the picture pyramid used in the CNN system in Figure 11.

The Laplacian pyramid is also used to segment medical images semantically, and the reader can refer to the article in [113]. Authors have a multiresolution redevelopment architecture based on a Laplacian pyramid that utilizes higher-resolution map jump connections and a polygonal gate to change reconstructed boundaries with low-resolution function maps gradually. Reference [114] introduces a method of scene interpretation, and, through the Laplacian pyramid, the raw input image is transformed. In comparison, CNN creates a series of feature charts and generates each scale in two phases.

### 5.2. Atrous Spatial Pyramid Pooling (ASPP)

**Figure 12:**
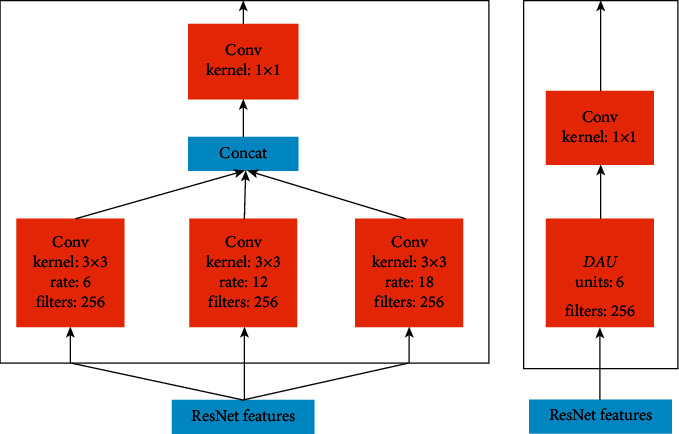
ASPP architecture (distance in convolution is not shown as the real rate in this image) [15].

Inspired by the image pyramid technique, [92] Atrous Spatial Pyramid Pooling (ASPP) is suggested to be done to provide robust object segmentation at different scales. ASPP explores the powerful fields-of-views (FOV) and the convolution feature layer with a multisampling rate filter and then captures the artifacts at various scales in the scene. ASPP architecture is seen in Figure 12.

### 5.3. Pyramid Pooling

**Figure 13:**
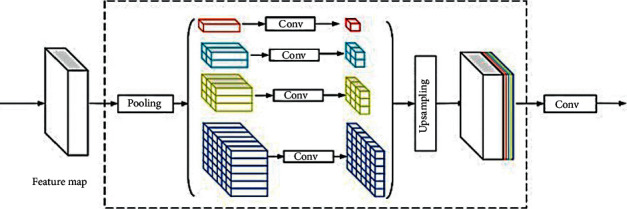
Demonstration of the structure of a pyramidal pooling [116].

According to the Pyramid Pooling shown in Figure 11, through gathering image data based on various regions, [115] exploits global knowledge capacity and calls its pyramid scene parsing network, known as PSPNet. The excellent results therein show that, with pyramidal pooling, a PSPNet brings a new mIoU score record of 85.4 percent in PASCAL VOC 2012 and brings 80.2 percent in Cityscapes dataset by experiments in [115]. Pyramid Pooling adopts multiple pooling size scales and applies the output to the original size for sampling processing. Finally, to shape a composite feature profile, it obtains the findings. Different scales of the size of the pool with different colors are marked in Figure 13. In general, pyramidal pooling can be used for any mapping of features. For example, the program in [115] applies pyramidal pooling in the pool5 layer.

### 5.4. Feature Pyramid

As mentioned in research backgrounds such as [117], the feature pyramid is an important component in image work for recognizing objects of various sizes. Object detectors have avoided displaying pyramids with recent DL methods because the computational volume and memory are compact. In [117], authors use the CNN multiscale pyramid hierarchy to build special pyramids at an additional cost. Also, a Feature Pyramid Network (FPN) has been created to construct high-level semantic maps at all scales. Machine learning also has many applications in the optimal selection of feature extraction [118–122].

## 6. Multilevel and Multistep Feature Methods

**Figure 14:**
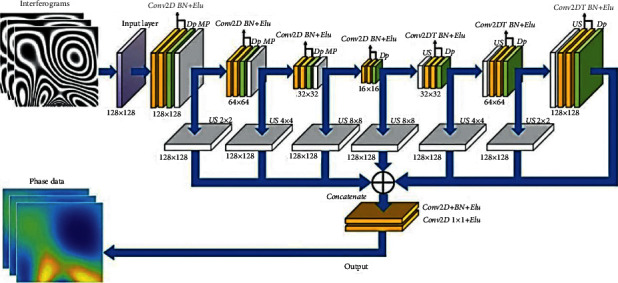
The structure adopted as hypercolumns [124].

CNN may be known as a feature extractor [123], and, as a feature, CNN-based detection algorithms usually use the last-layer output. For dense forecasting, however, the data in this layer is too big. Instead, in localization, the primary layers can be correct but do not present the meaningful state. They describe hypercolumns as the activation vectors of all CNN units above that pixel to achieve both. You will see the form adopted as superstores in [123] in Figure 14. The FCN [86] has already approved jumps, as seen in Figure 5. The multilevel approach appears to have been used in the study [123], and multimodeling is a group approach to visualization ([125, 126]). In comparison to the multilevel approach, the multistage technique is utilized in semantic segmentation [107] to improve its accuracy and speed, which recommends the deep layer cascade (LC) method. The deep layer cascade (LC) method consists of multiple independent models, unlike the conventional model cascade (MC) ([125, 126]). As a multisubset model cascade, the LC system uses a single deep model, classifying several basic parts into the shallow stage and concentrating the deeper stage on several hard sections. This not only increases the productivity of segmentation but also accelerates both deep network training and research (Figure 12).

## 7. The Most Practical Deep Learning Methods in Medical Image Segmentation

**Figure 15:**
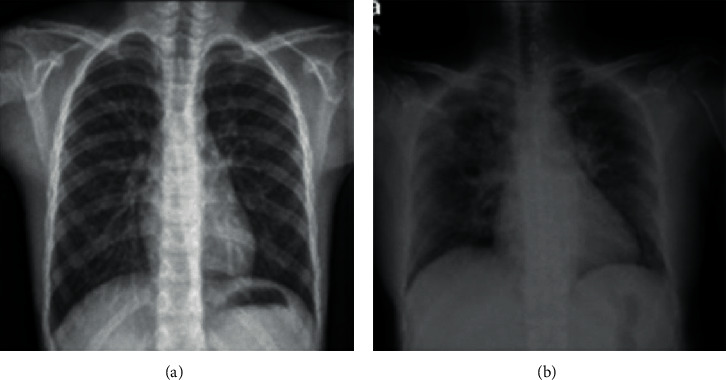
(a) Radiographic images of a healthy person's chest. (b) Chest radiographs of patients with COVID-19 [143].

The application of DL techniques to segmentation methods and MRI imaging aiming at brain tumors has been studied extensively. In general, different structures of a convolutional neural network can be acknowledged as the best DL technique in this research with studies on a convolutional neural network with a deeper layer [127], two-way convolutional neural network [128], cascaded CNN [129], multidimensional convolutional neural network [130], fully convolutional neural network (FCNN) for training with CRF [131], three-dimensional model of the convolutional neural network, two-dimensional model of the convolutional neural network [132], extreme learning machine (ELM) [133], Growing Deep Convolutional Network (GCNN) [134], complete convolutional neural network with Atheros convolution pyramid features [135], three-dimensional convolutional neural network test-time augmentation [136], and convolutional neural network referred to as CRF-based multicascade [137]. All the weaknesses along with the general application of all the advantages of the classical methods are presented in Table 2, and it is seen in the available and studied methods and also all of them have a wide range of applications. Yang et al. have used a portable evanescent wave sensor to detect SARS-CoV-2 using a CRISPR-based [138]. Reference [139] also uses DL methods to detect and classify breast tumors. Three different DL architectures, GoogLeNet, VGGNet, and ResNet, have been considered, and analysis has been performed between these methods. Visual detection and evaluation of breast tumors with DL principles are also presented in [140], which uses the combined methods of K-means and SURF algorithms in the structure of DL networks based on multiclass support vector machine. The detection of breast cancer using an extreme learning machine (ELM) based on feature fusion with deep convolutional neural network features is presented in [141]. Also, in [142], the extraction of a distinct pattern for the histopathological image classification of breast cancer has been done through an automated structure based on a convolutional neural network. All the weaknesses along with the general application of all the advantages of the classical methods are presented in Table 3; and it is seen in the available and studied methods and all of them have a high range of applications.  Figure 15 shows chest radiographs in healthy individuals and COVID-19 patients, respectively (see Table 4).

## 8. Discussion

A primary application in image processing and computer vision is image medical semantic segmentation. In addition to a brief overview of image semantic segmentation and traditional medicine, this article discusses recent advances in image semantic segmentation, particularly based on deep convolutional neural networks in the following aspects: (1) fully convolutional network, (2) method of sampling method, (3) combining FCN with CRF methods, (4) dilated convolution approaches, (5) progress in the main pillar of networks, (6) pyramid methods, and (7) multistage properties and multilevel methods. So far, there have been more and better ways to segment medical images semantically more accurately or faster or both with higher accuracy and speed, as well as better performance. Finally, the authors of this article hope that this review of recent advances in image medical semantic segmentation will help researchers in this field.

Maghdid et al. [21] reviewed a comprehensive, preprocessed dataset on X-rays and CT scan images from a variety of sources and provided an algorithm for accurate diagnosis of COVID-19 using DL and transmission learning tools. Also, a modified model was used by CNN and AlexNet as a pretested network on ready-made X-ray and CT scan datasets. After extensive experiments in both datasets, it has been shown that the proposed COVID-19 model predicts high accuracy and low response time. It is important to note that their proposed DL pattern has shown equivalent performance compared to that of a specialist radiologist. In addition, it can significantly improve the efficiency of radiologists while performing clinical practice [21]. Researchers are searching for new ways of screening, and the DL added to the chest X-rays of patients has shown positive outcomes. The computational cost of these approaches is still high considering their popularity, which causes difficulties with accessing them. Therefore the main purpose of this research is to accommodate the COVID-19 screening issue in chest X-ray with a reliable and successful approach in terms of memory and processing time. DL is a branch of artificial intelligence (AI) machine learning related to algorithms that are inspired as artificial neural networks by the structure and operation of the brain by using far higher-quality input images without any processing time. In addition, it is faster and cheaper to embed these versions in devices with more limited settings such as smartphones. To make use of embedded and large-scale devices, models can need little memory and carry out research quickly; and it encourages smartphones and emergency devices to work with them. DL models are complicated, so, to avoid inserting connections, a large number of things are necessary. In the training suite, for example, where the learning network performs well, to have less performance in the test suite, a large number of items are required. Unfortunately, there is not much data available for most real-world problems, even though the dataset is still small. Efficient training in deep neural networks has also been rendered possible through studying data transmission and amplification strategies in the small number of COVID-19-related images [144]. A popular approach for survival analysis and event prediction is the CPH model. This is therefore a semiparametric model, which suggests that the probability of misdiagnosis is a linear mixture of the clinical variables of the patient. In a fully data-driven way, the DL model can learn and infer high-order nonlinear interactions between clinical variables and disease effects. Data improvement techniques in DL will also make the model more robust to information noise and lost information, which usually happens in clinical datasets. It is also possible to expand the DL model to incorporate time-dependent factors such as vital signs and elevated visual attributes such as CT or X-ray images. It is inevitable to lose data on certain factors in reality and the real world. Data lost in less than three variables was then permitted in authors' online measurement tool, and risk evaluation based on DL methods can still be given by the field. In the clinical experience of Liang et al. [152], mild cases of COVID-19 are generally limited, and these are acute cases that need to be further investigated by physicians. Classified cases of their patients are clinically and economically expensive to manage COVID-19, especially due to the rapid outbreak of the disease which can happen and the high mortality rate related to acute disease, which has a high cost. By submitting clinical information online, medical personnel can use the predicted risk index to hospitalize patients and accordingly arrange patient treatment plans. In this way, medical resources can be appropriately allocated [152]. Arora et al. [26] suggested a DL model for estimating the number of patients who may have COVID-19 infection. They estimated the number of new cases of new coronaviruses in various states of the Indian Union for a span of one day to one week. For prediction, they utilized repetitive neural networks and Long Short-Term Memory (LSTM) and then tested several LSTM models in the Indian dataset and concluded that deeper LSTM models such as stacked LSTM, circular LSTM, and two-way LSTM were more accurate than simple LSTM models. To date, no research studies on COVID-19 cases have been reported from all Indian states, according to the authors. In one study, to predict the number of COVID-19-positive cases in the Indian states, Arora and colleagues suggested DL models. Because of the growing number of positive cases in India, exploratory data analysis has been undertaken. Depending on the number of cases and the daily growth rate, the government classifies states into mild, moderate, and severe areas to take strong action against the quarantine of the entire country, and this may cause economic and social problems. As predictive models, recurrent neural networks (RNN) are used based on long-term and short-term memory (LSTM) cells. In 32 states/union states, LSTM types such as deep LSTM, circular LSTM, and bidirectional LSTM models have been tested, and the model is chosen with maximum accuracy based on absolute error. Based on estimation errors, the best outcome is the two-way LSTM, and the worst result is the hanging LSTM [26]. Ardakani et al. [149] suggested a DL-based CAD method to classify COVID-19 versus other pneumonic and abnormal pneumonia in research. They proposed that the DL method will assist radiologists to diagnose the disease associated with COVID-19, and they used ten convolutional neural networks (CNNs) to identify COVID-19-related diseases. Ten well-known CNNs were used in this analysis to provide a detailed view of the role of artificial intelligence in COVID-19 diagnosis. Data have shown that DL can distinguish COVID-19 with high accuracy from other pneumonia and viral diseases. For the ResNet-101 and Xeption networks, the best findings have been found. In the classification of COVID-19 and non-COVID-19 diseases, however, the Xeption network was most successful, but it did not have the highest sensitivity. In comparison, ResNet-101 was able to detect COVID-19 infection with the highest sensitivity and present fewer features compared to the Xeption network. In diagnosing patients with COVID-19, the trend is to incorporate a system with the greatest sensitivity. The advancement of DL programs helps researchers to do fast and deep X-ray scan analysis. DL is a mixture of methods of machine learning that focuses primarily on the automated extraction and classification of image characteristics, while its applications are commonly employed in medical work, medical detection, and classification. Machine learning and DL in the application of artificial intelligence for mining, pattern analysis from data, have been created as a discipline. To further evaluate the deep cognition method, Apostolopoulos et al. performed an experiment using six common lung diseases, including COVID-19. In this method, its capabilities in differentiating between different diseases are evaluated. Fine-tuning a deep network, in the context of DL, is a common approach for both learning the properties of depth and maintaining the method for extracting global properties, which exist in each image as different shapes. Specific research to detect potential trademarks focuses on X-ray images, and these biomarkers can be substantially correlated with COVID-19 disease. However, DL derives from images a large range of high-dimensional features, and some of these features may be known as real image markers. Li et al. studied the effect of self-assembly on fluorescent in magnetic fluid flow and its use for a new COVID-19 detection [149]. Recently, many studies have been done on various subjects about COVID-19, such as scheduling problems [153], climate change [154], sunspot assessment [155], disease severity and industry [156], energy after COVID-19 pandemic, travel-related risks among pandemics [157], and predictive modeling [148].

## 9. Limitations

The limitations of this research are mentioned in several aspects. First, the CT validation dataset is collected at one center, which may not represent all COVID-19 patients in other geographic areas. The generalization of the DL system must be approved in several centers. Second, the system is designed to determine the outbreak of the disease and may not be effective in measuring other pneumonia, such as bacterial pneumonia. Finally, in the next work, the authors will develop a system for quantifying the total intensity of pneumonia using transfer learning. Not ready for production, the researchers hope that the results obtained by COVID-Net in the COVIDX test dataset will be available as open source with descriptions of the open-source dataset. CXR images were used to accelerate the development of high-precision DL solutions for the diagnosis of COVID-19 patients. The future work will continue to include increasing accuracy and PPV for COVID-19 with the collection of new data, as well as the development of COVID-Net for risk classification for survival analysis, patient status prediction, and length of hospital stay.

## 10. Conclusion

In this study, the potential of deep learning methods in COVID-19 diagnosis is investigated. This study has reviewed the classification systems based on DL to assess the extent of the disease. This system not only automatically contours the infected areas but also measures their shape, volume, and percentage of infection on a CT scan of patients with COVID-19. The methods involve radiologists to intervene effectively in the results of DL segmentation and repeatedly add more tutorials to update the model, thus accelerating the algorithm's development cycle. CT imaging has become an effective tool for screening patients with COVID-19 and for assessing COVID-19 levels. However, radiologists do not thave a computer tool to accurately determine the severity of COVID-19, for example, the percentage of infection in the lungs. DL has become a common method in medical image analysis and has been used in the analysis of lung diseases. Using this deep learning automated segmentation, many studies on imaging quantification and its association with syndromes, epidemiology, and therapeutic responses can provide further information on improving the diagnosis and treatment of COVID-19. An AI algorithm can be created quickly from one or more algorithms that do the same thing. This is in contrast to the standard method for generating a DL algorithm, which requires several steps. In order to review the data, expert annotations are needed at the data collection point at which a large number of samples need to be taken. The second is the process of training in which the data obtained is used to train network models. Every category should be well represented so that the training can be generalized during the test process to the new objects found by the network. A great number of network parameters (typically in the order of millions) are created automatically in this learning process. The third step is the experiment in which the network is presented with another collection of objects not included in the testing and the network performance is statistically evaluated to determine its classification. There is no solution that fits all; we hope that the positive results obtained by COVID-Net will be present in the COVIDX test dataset. Images are used to boost the advancement of highly accurate DL solutions for the diagnosis of COVID-19 patients and accelerate the treatment of patients. Future pathways including continuing to enhance sensitivity and PPV to COVID-19 disease by collecting new data as well as extending the suggested COVID-Net to risk classification for analysis, patient status prediction, and length of hospital stay will be useful. [158–164]

## Figures and Tables

**Table 1:** Comparison between existing methods for semantic segmentation.

Reference	Year	Method	Advantages	Disadvantages
Bourdev et al. [28]	2010	HOG	Ability to specify areas, especially at the edges, with high clarity and accuracy	High computational complexity, inability to be used in a wide range of applications
Xia et al. [27]	2005	HOG	Ability to specify areas, especially at the edges, with high clarity and accuracy	High computational complexity, inability to be implied in an extended variety of applications
Lowe [29]	2004	SIFT	Accurate identification of areas and edges	High computational complexity, inability to be implied in an extended variety of applications
He and Wang [30]	1990	LBP	High capability in cryptography and image data encryption and edge detection operations	The algorithm is slow
Bay et al. [31]	2008	SURF	Accurate identification of areas and edges	High computational complexity, inability to be used in a wide range of applications
Derpanis [32]	2004	Harris corner detection	Ability to find the corners of an image outside the edges, ability to be used in a wide range of high-sensitivity image processing systems	Low accuracy and slow method
Shi and Tomasi [33]	1994	Shi-Tomasi	Ability to specify areas, especially at the edges, with high clarity and accuracy	High computational complexity, inability to be used in a wide range of applications
Medioni and Yasumoto [34]	1987	Corner detection with subpixels	Ability to find the corners of an image outside the edges, ability to be used in a wide range of high-sensitivity image processing systems	Low accuracy and slow method
Smith and Brady [35]	1997	SUSAN corner detection	Accurate edge detection based on texture and brightness and better capabilities than classic operators such as Canny and Prewitt	Low accuracy in high-resolution images and slow method
Rosten and Drummond [36]	2005	FAST	Ability to specify areas, especially at the edges, with high clarity and accuracy	High computational complexity, inability to be used in a wide range of applications
Rosten et al. [37]	2010	FAST-ER	Ability to specify areas, especially at the edges, with high resolution and precision in multiscale modes	High computational complexity, inability to be used in a wide range of applications
Mair et al. [38]	2010	AGAST	Ability to specify areas, especially at the edges, with high clarity and accuracy	High computational complexity, inability to be used in a wide range of applications
Leutenegger et al. [39]	2011	Multiscale AGAST	Ability to specify areas, especially at the edges, with high resolution and precision in multiscale modes	High computational complexity, inability to be used in a wide range of applications
Venegas-Barrera and Manjarrez [40]	2004	BOW	Ability to specify areas, especially at the edges, with high clarity and accuracy	High computational complexity, inability to be used in a wide range of applications
Brox et al. [41]	2011	Poselets	Ability to specify areas, especially at the edges, with high clarity and accuracy	High computational complexity, inability to be used in a wide range of applications
Zhu et al. [42]	2005	Textons	Ability to specify areas, especially at the edges, with high clarity and accuracy	High computational complexity, inability to be used in a wide range of applications
Strauss and Hartigan [47]	1975	K-means	Ability to find clusters in images and cluster them	Slow method and need to combine with faster methods
Shen et al. [54]	2004	Edge detection and regional growth	Ability to specify areas, especially at the edges, with high clarity and accuracy	High computational complexity, inability to be used in a wide range of applications
Shan et al. [55]	2004	SVM	High capability in high-precision image classification operations in pairs and the ability to separate features with vectors	High computational complexity, slow method
Shotton et al. [56]	2006	MRF	High capability in high-precision image classification operations in pairs and the ability to separate features with vectors	High computational complexity, slow method
Hassantabar et al. [61]	2020	CNN	Ability to diagnose the COVID-19 infected lung tissue for segmentation and classification of patients	(i) Small numbers of images (ii) Unable to find illness severity
Dorosti et al. [62]	2020	Sensitivity analysis	This approach can help in the identification of beneficial parameters as well as the avoidance of patient mortality in all sorts of disease	(i) Data limit (ii) Ignoring other variables
Sharifi et al. [63]	2021	CNN	Diagnosis of fatigue foot using CNN	(i) High computational complexity (ii) Integrated only on CNN method
Laradji et al. [64]	2021	Supervised consistency learning	The best loss function for prediction	(i) Unable to detect patients uniquely (ii) Should be connected to other methods

**Table 2:** Comparison between existing methods in the segmentation field in MRI images.

Reference	Year	Method	Advantages	Disadvantages
Darwiesh et al. [57]	2016	The method of Brownian motion of water molecules to produce contrast	Detecting edge areas to separate sections with tumors and nontumor sections	(i) Lack of detection of tumors in other tumors or other areas (ii) High computational complexity and slow method (iii) Lack of separation of areas with benign and malignant tumors
Aslam et al. [58]	2015	Edge detection	Detecting edge areas to separate sections with tumors and nontumor sections	(i) Lack of detection of tumors in other tumors or other areas (ii) High computational complexity and slow method (iii) Low accuracy (iv) Lack of separation of areas with benign and malignant tumors
Qiao et al. [59]	2021	Watershed and hierarchical clustering algorithm	Detecting edge areas to separate sections with tumors and nontumor sections	(i) Lack of diagnosis of tumors in other tumors or other areas (ii) High computational complexity and slow method (iii) Low accuracy (iv) Lack of separation of areas with benign and malignant tumors
Ain et al. [60]	2014	Concrete anisotropic emission based on group classification, support vector machine (SVM), and FCM	High accuracy in diagnosing and classifying areas with tumors	Lack of comparison with previous methods and lack of consideration for comparison with DL methods or other neural networks
Mobahi et al. [48]	2011	Genetic algorithm and discrete wavelet transform threshold method	Detecting edge areas to separate sections with tumors and nontumor sections	
Karnan, and Selvanayaki [65]	2010	The combined approach of ant colony optimization algorithms and genetic algorithm	Detecting edge areas to separate sections with tumors and nontumors sections	(i) Lack of diagnosis of tumors in other tumors or other areas (ii) Very high computational complexity and slowness of the method (iii) Low accuracy (iv) Lack of separation of areas with benign and malignant tumors
Ghosh et al. [66]	2018	FCM-based chaotic firefly algorithm	(i) Detecting edge areas for separating tumor and nontumor sections (ii) High execution speed with the complexity of the method (iii) Accurate detection of features	(i) High computational complexity (ii) Lack of separation of areas with benign and malignant tumors
Zhu et al. [67]	2018	Particle swarm optimization (PSO)	(i) Detecting edge areas for separating tumor and nontumor sections (ii) High execution speed with the complexity of the method (iii) Accurate detection of features	(i) Lack of diagnosis of tumors in other tumors or other areas (ii) High computational complexity and slow method (iii) Lack of separation of areas with benign and malignant tumors
Alagarsamy et al. [68]	2019	Bat algorithm	(i) Detecting edge areas for separating tumor and nontumor sections (ii) High execution speed with the complexity of the method (iii) Accurate detection of features	(i) Lack of detection of tumors in other tumors or other areas (ii) High computational complexity and slow method (iii) Lack of separation of areas with benign and malignant tumors
Memiş et al. [158]	2020	Deep CNN	Finding the head bone femoral and femur properties for low-quality MRI images	(i) Small volume of the dataset for validation and verification (ii) Unable to support any types of disease
Duran et al. [159]	2020	Self-attention model	End-to-end attention model with multiple classes	(i) Only unable to detect prostate cancer (ii) An additional mechanism for CAD models
Hu et al. [137]	2019	3D-DenseUNet-569	(i) Adaptable to depthwise separable convolution (ii) Drop the GPU processing time	(i) Low-level feature extraction (ii) Improper for big data (iii) Unable to adapt to 2D images
Karayegen & Feyzi [161]	2021	Deep learning models	(i) High prediction method (ii) Differing modality of MRI images (iii) 3D image analysis	(i) Limited dataset for verification (ii) Do not use all image area (iii) Needs ground truth
Ahmadi et al. [162]	2021	Deep spiking neural network	(i) Low computational complexity (ii) Used quantum filter (iii) High accuracy	(i) Multistep method (ii) Overfitting in some analysis
Ahmadi et al. [163]	2021	Robust PCA and CNN	(i) Clustering and segmentation method (ii) Automated clustering (iii) Used remove outliers (iv) High accuracy and sensitivity	(i) High complexity (ii) Do not support 3D images

**Table 3:** Comparison between existing methods in the field of segmentation in mammographic images.

Reference		Method	Advantages	Disadvantages
Rouhi et al. [69]	2015	Regional growth with a cellular neural network with a specific threshold	Ability to diagnose benign and malignant tumors, high accuracy in classification	High computational complexity (i) Lack of accurate detection of areas with tumor (ii) Lack of comparison with previous methods
Kaymak et al. [70]	2017	Back-Propagation (BP)	A convenient way to use in neural network training, highly fast execution speed in training	Uncertainty of the exact type of approach proposed and lack of comparison at the time of classification and uncertainty of benign and malignant tumors
Karabatak [71]	2015	Naïve Bayesian	Ability to diagnose benign and malignant tumors, high accuracy in classification	High computational complexity (i) Lack of accurate detection of areas with tumor (ii) Lack of comparison with previous methods
Wang et al. [72]	2018	Regression-based methods	Ability to estimate and predict remaining life based on tumor size, high accuracy in detection	
Pereira et al. [73]	2014	Wavelet analysis and genetic algorithm	Ability to diagnose benign and malignant tumors, high accuracy in classification	High computational complexity (i) Lack of accurate diagnosis of areas with tumor (ii) Lack of comparison with previous methods
Cordeiro et al. [75]	2016	Semisupervised adaptive algorithm GrowCut	Ability to diagnose benign and malignant tumors, high accuracy in classification	High computational complexity (i) Lack of accurate detection of areas with tumor (ii) Lack of comparison with previous methods
Ahmed et al. [76]	2020	Mask RCNN	(i) Increased AUC for transfer learning (ii) Use for X-ray mammographic image	(i) Low accuracy (ii) Used low volume dataset for verification (iii) High rate of oversampling
Lee et al. [77]	2020	Multiscale grid average pooling	(i) Utilizing global and local spatial feature (ii) Novel attention module (iii) Ultrasound image dataset	(i) Lower accuracy of segmentation (ii) High computational complexity (iii) A small volume of the dataset
Soulami et al. [78]	2021	UNet model	(i) High accuracy for breast cancer detection (ii) High f1-score and AUC	(i) High complexity model (ii) Overfitting in some models (iii) Lower volume of analysis
Huang et al. [79]	2021	Fuzzy fully CNN	(i) Fuzzy membership function (ii) Conditional random fields	(i) Low sensitivity (ii) Low intersection over union (iii) Low resolution and poor quality

**Table 4:** DL methods used in COVID-19 detection and diagnosis.

Author	Purpose	Method	Advantages	Disadvantages	DL architecture	Results
Luz et al. [144]	To provide an accurate and efficient method for COVID-19 screening with chest X-rays for memory and processing time	Using EfficientNet artificial neural networks	High accuracy	Large and heterogeneous database	The use of algorithms, or artificial neural networks, is inspired by the brain's structure and function	From the hierarchical classification, experiments were performed to evaluate the performance of the neural network in the COVID-19 data. It became possible to use data transfer techniques and reinforce data. The accuracy was 93.9
Liu et al. [12]	Determining areas of infection and examining the lungs with the help of a chest CT scan	Use of CT scans to evaluate COVID-19, evaluation of system performance based on DL. This experiment was performed on 249 patients	Patient availability, high accuracy	Lack of sufficient information	Classification based on DL of VB-Net neural network	A DL system was developed for the segmentation and measurement of infection areas in CT scans of patients with COVID-19. The quantitative evaluation showed high accuracy for the infected area based on POI criteria
Li et al. [13]	Development of artificial intelligence CT imaging tools to diagnose coronavirus and isolate sick people away from healthy people	Uses powerful 2D and 3D DL models. Modifies and adapts existing AI models. This experiment was performed on 157 patients	High accuracy	Complexity	2D and 3D DL models were used	The AI-based analysis is rapidly evolving in the diagnosis of coronavirus, and the detection is being made with great accuracy
Wang and Wong [14]	Assist physicians in improving COVID-19 screening	Polymer reverse chain reaction screening from RT-PCR to diagnose COVID-19	Better understanding and character analysis by physicians, accelerating the development of high-precision DL solutions	Complexity, being time-consuming	Use of artificial intelligence systems based on DL, hardening and evaluation of COVID-Net prototype using Keras DL library with TensorFlow background	Assist physicians in improving screening, use of CXR images to diagnose COVID-19
Ghoshal and Tucker [145]	Evaluation of prop weights-based elliptic irritable neural networks (BCNN) to improve performance for COVID-19 diagnosis	Using the transmission learning method in COVID-19 X-ray images	Improvements in the diagnosis of COVID-19	Uncertainty in detection for radiologists	Use of DL for classified tasks, as well as chest radiographic diagnosis for COVID-19	Estimated uncertainty with DL can warn radiologists of incorrect predictions, which increases the use of DL in diagnosing the disease
Narin et al. [146]	Evaluation of the use of focal neural network-based methods to detect an infected patient using X-ray radiography of the chest	Use InceptionV3, ResNet50, and InceptionResNetV50 to diagnose infected patient	High-performance ResNet50 model (i) High accuracy (ii) Cost reduction	Ambiguity in matrices	Using COVID-19 X-ray images for DL models, using transition learning methods	Preliminary diagnosis of COVID-19 patients to prevent the spread of this disease in other people, using the ResNet50 model with 98% accuracy
Hemdon et al. [20]	The implementation of a DL framework for automated COVID-19 diagnosis of X-ray images	It was performed on 50 chest X-ray photographs of 25 positive COVID-19 cases. Seven distinct architectures from deep concealer neural network models are used in COVIDX-Net	Automatic diagnosis of COVID-19	Complexity	Use the COVID-19 classification to diagnose COVID-19 on X-ray images automatically using one of the DL frameworks	X-ray images based on the COVIDX-Net framework proposed
Zhang et al. [147]	Detection and differentiation of viral patient from the nonviral patient	Experiment on COVIDX data including 106 COVID-19 cases	Strengthen and improve the model, more efficiency in treatment	Complex calculations	Analysis of medical images including staging detection and drawing of pathological abnormalities, X-ray image change	Detection of the abnormality by viral pneumonia screening works well on chest X-ray images The learning model is useful for predicting job failure We have the CAAD model and we have never seen such cases in COVID-19, the data had 83.61% AUC, and the sensitivity was 70.71%
Maghdid et al. [21]	Provide artificial intelligence tools for fast and accurate detection of COVID-19, create a comprehensive set of X prototypes	Using X-rays and scanning CT images and using DL algorithms	Creating intelligent detection methods with higher efficiency (i) Increasing detection speed (ii) Increasing accuracy	Complexity	Build a DL-based detection system to detect COVID-19 pneumonia using DL algorithms	Accelerate the diagnosis of COVID-19 using the CNN model
Zeraati et al. [148]	Automatic classification of lung diseases including COVID-19 with X-ray images	Use of advanced convolutional neural network called MobileNet, use of 3905 X-ray images of more than 6 patients	Automatic diagnosis of COVID-19 from medical images (i) Low cost (ii) High speed	Limitations	Use DL to extract large-sized features from medical images	Low-cost, fast, and automatic diagnosis of COVID-19. Different infections may be differentiated by computer and detection using features extracted by DL
Li et al. [149]	Provide a fast and reliable way to diagnose COVID-19	Use 1020 CT images of 108 patients with COVID-19, use of ten confidential neural networks to diagnose COVID-19	Rapid diagnosis of COVID-19, being valid	High expenses	Use of CAD system based on DL to classify COVID-19 against other pneumonia	Using the textual CAD method on CT images to differentiate COVID-19 from other pneumococcal diseases. ResNet-101 can be used to diagnose COVID-19
Arora et al. [26]	Predict the number of new coronaviruses	Use LSTM-based RNN for forecasting	High accuracy of forecasting	High complexity and volume of data	Use the LSTM DL model	Two-way LSTM gives the best result and confidential LSTM gives the worst result. biLSTM gives very accurate results for short-term forecasts, such as 1 to 3 days, with less than 3% error
Huang et al. [150]	Quantitative evaluation of changes in lung tolerance in patients with COVID-19 using CT scan with an automated DL method	CT images show the entire lung and are measured and compared by commercial DL software	Classification of different groups and better detection	Loss of initial findings	Chest CT image evaluation using DL	The lung failure rate in COVID-19 was measured using a DL instrument based on a chest CT image and there was a significant difference between different groups
Oh et al. [151]	Use of the neural network to diagnose COVID-19	Inspired by CXR radiographic imaging	The usefulness of this method for the diagnosis of COVID-19 and patient triage	Difficulty in training deep neural network, difficulty in collecting big data	Use of X-ray chest images to classify COVID-19	Use artificial intelligence to improve CXR performance for detection
Liang et al. [152]	Use of a DL model to predict disease	Inclusion of 1590 patients from 575 medical centers, Use of DL models	Early detection	Complexity of calculations	Use of an integrated Cox model called Survival Cox DL	At least 60% of the data were used for prediction. A DL model was used to predict which was efficient

## Data Availability

This is a review paper and data sharing is not applicable.
